# Cerebral reactivity in migraine patients measured with functional near-infrared spectroscopy

**DOI:** 10.1186/s40001-015-0190-9

**Published:** 2015-12-08

**Authors:** Ahmadreza Pourshoghi, Arash Danesh, David Stuart Tabby, John Grothusen, Kambiz Pourrezaei

**Affiliations:** School of Biomedical Engineering Science and Health, Drexel University, Room 131, 3508 Market St, Philadelphia, PA 19104 USA; Neurology Department, Drexel University College of Medicine, Philadelphia, USA; Optimum Neurology, Bala Cynwyd, USA

**Keywords:** Functional near-infrared spectroscopy, Vascular theory of migraine, Pain assessment, Migraine, Cerebrovascular reactivity

## Abstract

**Background:**

There are two major theories describing the pathophysiology of migraines. Vascular theory explains that migraines resulted from vasodilation of meningeal vessels irritating the trigeminal nerves and causing pain. More recently, a neural theory of migraine has been proposed, which suggests that cortical hyperexcitability leads to cortical spreading depression (CSD) causing migraine-like symptoms. Chronic migraine requires prophylactic therapy. When oral agents fail, there are several intravenous agents that can be used. Understanding underlying causes of migraine pain would help to improve efficacy of migraine medications by changing their mechanism of action. Yet to date no study has been made to investigate the link between vascular changes in response to medications for migraine versus pain improvements. Functional near-infrared spectroscopy (NIRS) has been used as an inexpensive, rapid, non-invasive and safe technique to monitor cerebrovascular dynamics.

**Method:**

In this study, a multi-distance near-infrared spectroscopy device has been used to investigate the cortical vascular reactivity of migraine patients in response to drug infusions and its possible correlation with changes in pain experienced. We used the NIRS on 41 chronic migraine patients receiving three medications: magnesium sulfate, valproate sodium, and dihydroergotamine (DHE). Patients rated their pain on a 1–10 numerical scale before and after the infusion.

**Results:**

No significant differences were observed between the medication effects on vascular activity from near channels measuring skin vascularity. However, far channels—indicating cortical vascular activity—showed significant differences in both oxyhemoglobin and total hemoglobin between medications. DHE is a vasoconstrictor and decreased cortical blood volume in our experiment. Magnesium sulfate has a short-lived vasodilatory effect and increased cortical blood volume in our experiment. Valproate sodium had no significant effect on blood volume. Nonetheless, all three reduced patients’ pain based on self-report and no significant link was observed between changes in cortical vascular reactivity and improvement in migraine pain as predicted by the vascular theory of migraine.

**Conclusion:**

NIRS showed the potential to be a useful tool in the clinical setting for monitoring the vascular reactivity of individual patients to various migraine and headache medications.

## Background

Neural and vascular theories are proposed as the main theories describing the pathophysiology of migraines [1]. Traditionally, it was thought that migraine resulted from vasoconstriction of cranial blood vessels leading to compensatory vasodilation. More recently, consensus has shifted toward a neural theory of migraine which considers the excessive neocortical cellular excitability as the main cause [2].

There are a number of studies reported in the literature that suggest possible different cortical vascular responses between healthy individuals and migraine patients in different scenarios. In several of these studies, NIRS has been used as a non-invasive method of measuring cerebrovascular reactivity [3].

Akin et al. have shown that amplitudes of deoxygenated hemoglobin (Hb) and oxygenated hemoglobin (HbO_2_) signals acquired by NIRS are approximately two to five times higher in controls than migraine patients during four consecutive breath-holding tasks and concluded their results as a confirmation of an impaired cerebrovascular reactivity in the frontal cortex of migraine patients [4]. Shinoura et al. have compared changes in total hemoglobin (THb) and regional oxygen saturation (rSO_2_) of the right and left frontal lobes in response to intracranial pressure changes during the interictal period of migraine. According to their findings, the head-down maneuver resulted in a significantly smaller increase in right-sided total hemoglobin in migraineurs compared to volunteers. Moreover, it resulted in a small decrease in right-sided rSO_2_ and a significantly greater decrease in left-sided rSO_2_ in migraineurs compared to volunteers [5]. In another study, both TCD (transcranial Doppler) and NIRS were used in migraineurs without aura versus healthy subjects in a breath-hold challenge. Strong differences in the cerebral blood flow velocity (CBFV), a reduced increase of HbO_2_ and different hemoglobin balancing during breath-hold task have been reported for migraineurs. They have also concluded that migraineurs do not show marked vasodilation as a functional response to the CO_2_ increase [6]. The same parameters have been measured by Vernieri et al. using TCD and NIRS during carbon dioxide inhalation sessions of healthy subjects and migraineurs with aura (MA). Cerebral vasomotor reactivity (VMR), total hemoglobin content and percent oxygen increases were significantly greater on the predominant compared with the non-predominant migraine side. These findings suggest altered autoregulation in MA patients, possibly secondary to impaired cerebrovascular autonomic control [7].

In this study, we use NIRS to investigate the cerebrovascular effects of migraine medications and its possible correlation with patients’ pain improvement in real time.

In the remainder of this paper, we compare the vascular reactivity of 41 migraine patients receiving infusions of magnesium sulfate (21 patients), valproate sodium (12 patients) and dihydroergotamine (6 patients). We also correlate our findings with the subjects’ pain score self-reports. We aimed to investigate the utility of NIRS in comparing the analgesic efficacy of the above drugs based on patient self-reports compared with the measured cortical vascular reactivity in the clinical setting.

## Methods

### NIRS basics

Light in the near-infrared range (600–900 nm) penetrates the tissue because water, the key overall absorber of light in human tissue, has a low NIR absorption coefficient. In this range, tissue behaves as a turbid medium and light is significantly scattered by oxyhemoglobin (HbO_2_) and deoxyhemoglobin (Hb) molecules. There are other absorbents such as melanin and lipids; however, their overall average concentrations along the total optical path are negligible.

**Fig. 1:**
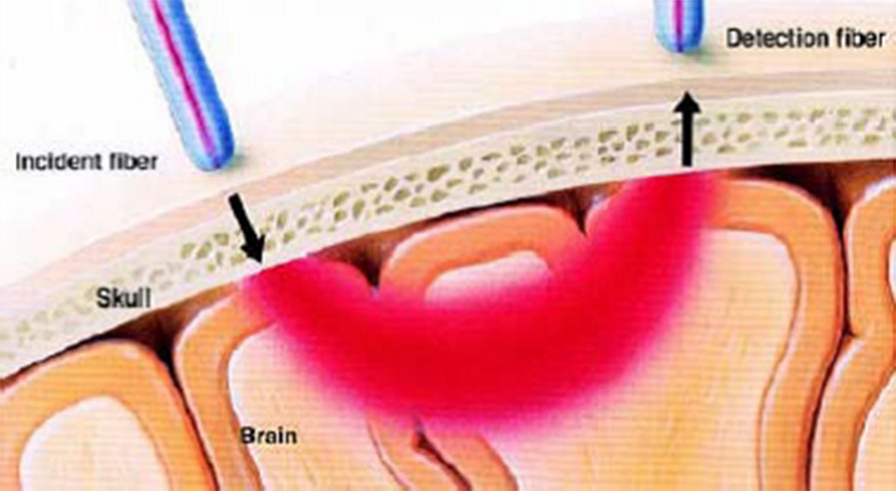
Volume of tissue sampled by an NIRS measurement: the highly scattered photons are reflected back to the tissue’s surface, mostly within a banana-shaped optical pathway. As a result, a photodetector placed on the skin (on the same surface as the source) can measure the reflected light [19]

As a result the NIR light detected in the Fig. 1 configuration is absorbed and scattered mainly by the oxyhemoglobin and deoxyhemoglobin molecules present in the blood stream. Any change in the concentrations of these molecules along the optical path of the detected photons will affect the detected signal. These changes can be the result of overall blood flow changes or local consumption of oxyhemoglobin due to neuronal activities.

Mathematical equations that govern this relationship are known as the Modified Beer–Lambert law [8]. The modified Beer–Lambert law states that changes in the concentration of light absorbing components are proportional to changes in light attenuation, divided by mean optical pathlength and extinction coefficients of the chromophores in the tissue. Optical pathlength is a measure of the average distance that light travels between the source and detector after several episodes of scattering and absorption.

The relative change in the concentration of Hb and HbO_2_ molecules can be calculated by the following equations:

1 $$\Delta \left[ {\text{HbO}_{2}} \right] = \frac{{\alpha_{\text{HbO2}} \left( {\lambda_{2} } \right){ \cdot }\frac{{\Delta A\left( {\lambda_{1} } \right)}}{{DP(\lambda_{1} )}} - \alpha_{\text{HbO2}} \left( {\lambda_{1} } \right){ \cdot }\frac{{\Delta A\left( {\lambda_{2} } \right)}}{{DP(\lambda_{2} )}} }}{{\alpha_{\text{Hb}} \left( {\lambda_{1} } \right){ \cdot }\alpha_{\text{HbO2}} \left( {\lambda_{2} } \right) - \alpha_{\text{Hb}} \left( {\lambda_{2} } \right){ \cdot }\alpha_{\text{HbO2}} \left( {\lambda_{1} } \right)}}$$

2 $$\Delta \left[ {\text{Hb}} \right] = \frac{{\alpha_{\text{Hb}} \left( {\lambda_{1} } \right){ \cdot } \frac{{\Delta {\text{A}}\left( {\lambda_{2} } \right)}}{{{\text{DP}}(\lambda_{2} )}} - \alpha_{\text{Hb}} \left( {\lambda_{2} } \right){ \cdot }\frac{{\Delta {\text{A}}\left( {\lambda_{1} } \right)}}{{{\text{DP}}(\lambda_{1} )}} }}{{\alpha_{\text{Hb}} \left( {\lambda_{1} } \right){ \cdot }\alpha_{\text{HbO2}} \left( {\lambda_{2} } \right) - \alpha_{\text{Hb}} \left( {\lambda_{2} } \right){ \cdot }\alpha_{\text{HbO2}} \left( {\lambda_{1} } \right)}}$$

### Experiment protocol

We studied three commonly infused medications: magnesium sulfate (MgSO_4_), valproate sodium, and dihydroergotamine (DHE). DHE is a potent vasoconstrictor. It may constrict meningeal blood vessels causing symptomatic improvement consistent with the vascular theory. Magnesium sulfate has vasodilatory properties [9, 10] through its effects on serotonin, but its main effects are thought to be neural. Low levels of magnesium sulfate are associated with disinhibition of NMDA receptors. Magnesium-mediated calcium influx to NMDA neurons results in inhibition. Magnesium sulfate ions block calcium influx and can prevent disinhibition. Low levels of magnesium lower the threshold for CSD (cortical spreading depression).

Another group of drugs which are widely used in migraine are sodium channel antagonists. Among these antiepileptic drugs, valproate sodium (Depacon) and topiramate seem to be more effective in migraine, as reported in the majority of controlled studies [11]. Valproate sodium has no major vascular role; it works through increasing GABA levels and in so doing suppressing CSD and migraines. Experimental evidence also shows that it suppresses neurogenic inflammation and attenuates nociceptive neurotransmission that leads to exacerbation of migraine.

These infusions are required when commonly used oral prophylactic drugs are not effective. Typically magnesium sulfate was given first, valproate sodium next and DHE was given last.

### Subjects

In this study, 41 patients (34 females) with an average age of 49.2 ± 9.5 years took part in a non-blinded trial using these medications; 21 subjects on magnesium sulfate, 12 subjects on valproate sodium and 8 subjects on DHE. Migraine subjects met the International Headache Society’s criteria for chronic migraine (ICHD-2) and were resistant to oral therapy. Patients rated their pain on a 1–10 scale (NRS-11) before and at the end of the infusion. The study was approved by the Drexel University Institutional Review Board (IRB) and informed consents and Ethics Committee approvals were obtained for all patients enrolled in the study, according to the Declaration of Helsinki.

### NIRS measurement

**Fig. 2:**
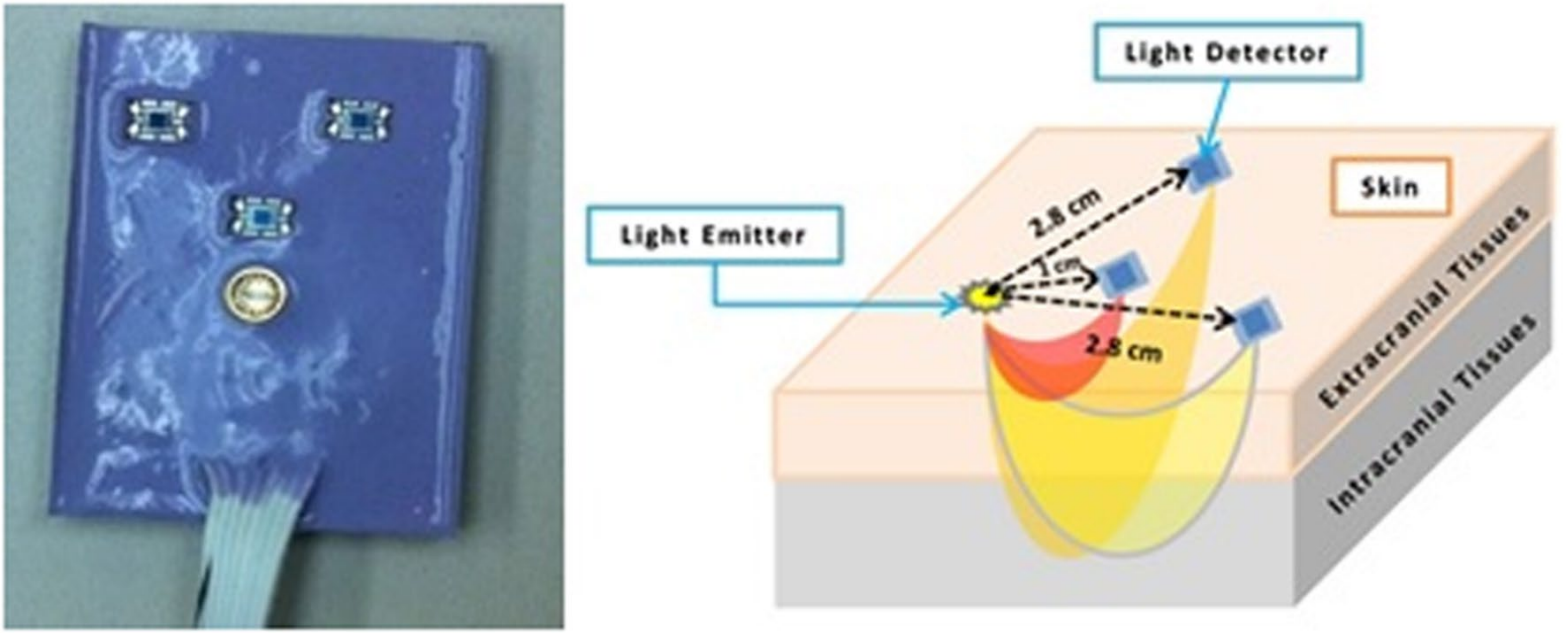
Configuration of NIRS probe: each probe has one LED source, one detector 1 cm from the source (near channel) and two detectors at 2.8 cm from the source (far channels)

Throughout the duration of the experiment, subjects were monitored by the NIRS probes that were attached to their foreheads. The probe configuration is shown in Fig. 2. Each probe is made of an LED source and three detectors. One detector is 1 cm from the source (near detector) and two detectors are 2.8 cm from the source (far detectors). The LED is a dual-wavelength LED (780 nm and 850 nm) manufactured by Epitex (L735/850-40D32) and the detectors are OPT101 manufactured by Texas instruments [12, 13]. Using the modified Bear–Lambert law (Eqs. 1, 2), the information about Hb and HbO_2_ is extracted from the raw intensity data.

**Fig. 3:**
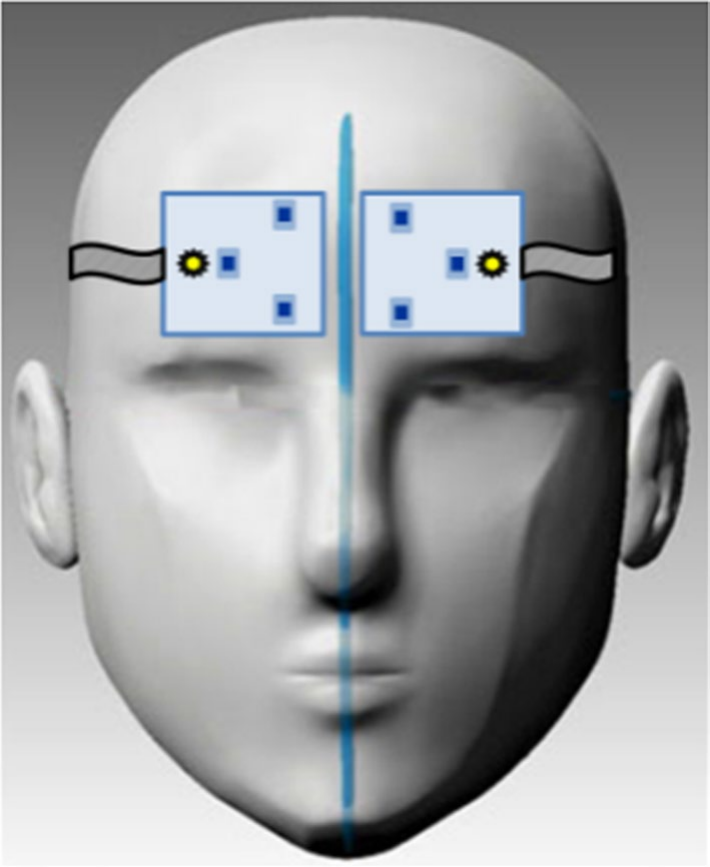
) NIRS probes placement: two NIRS probes are located on both sides of the forehead

After measuring the baseline for 2 min before infusion, we started the infusion and re-measured the cortical and superficial vascular activity with two NIRS probes. Probes were placed on the forehead of each subject—one on the right side of the forehead and one on the left side of the forehead and end of the probes were adjusted to align with the middle of the forehead—to cover position F_p1_, F_p2_ of the international 10–20 EEG system (Fig. 3).

**Fig. 4:**
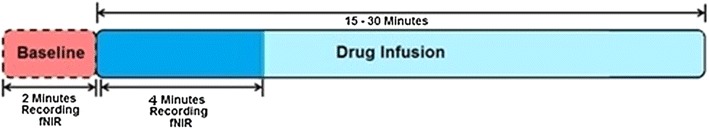
) Experimental Protocol: NIRS data recorded for 2 mins before and 4 min after start of infusion. Whole infusion takes 15–30 min

During this time, we ensured that all external factors that could have significant effects on the NIRS measurements were constant and controlled for; these include light, movement and temperature. Experimental protocol is shown in Fig. 4.

## Results and discussion

### Self-reported measures

**Fig. 5:**
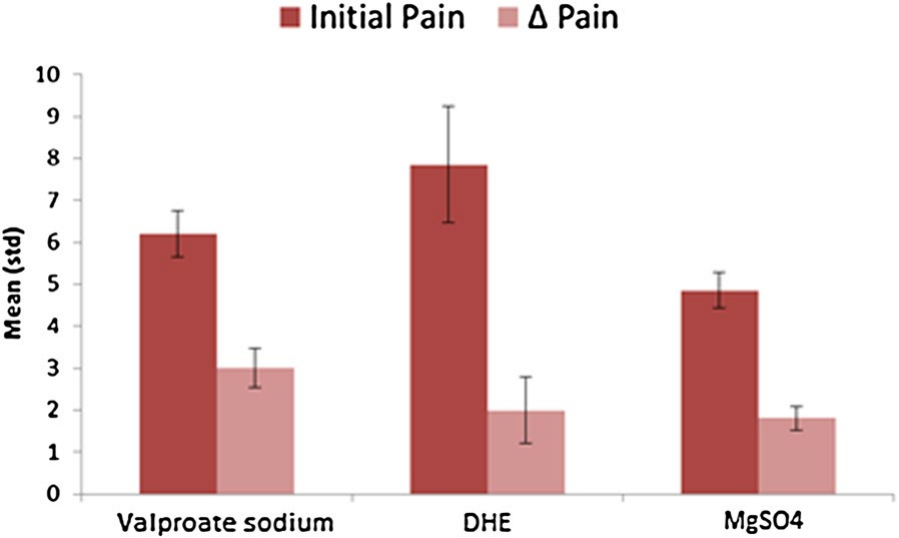
Self-reported pain scores by medication: subjects reported their pain based on a 1–10 numeric scale before and after the infusion. Changes between initial and final reported pain (Δ Pain) is considered as self-reported pain improvement

Patients rated their pain on a 1–10 scale (NRS-11) before and at the conclusion of the infusion. Figure 5 and Table 1 show initial pain and pain improvement after the infusion for each medication separately.

**Table 1:** Self-reported pain scores by medication: average initial pain and pain improvement have been shown for each medication as reported by subjects before and after infusion

Medication	Magnesium sulfate (n = 22)	Valproate sodium (n = 12)	DHE (n = 7)
Initial pain			
Mean	4.86	6.2	7.85
Standard deviation	2.05	1.97	3.93
Δ Pain			
Mean	1.81	3	2
Standard deviation	1.33	1.70	2.24

Reported initial pains were significantly different between medications (*P* ≤ 0.05) and were inherited from clinician’s policy of prescribing each drug. Average initial pain reported by patients for magnesium sulfate, valproate sodium and DHE was 4.4, 5.85 and 7.5, respectively. Magnesium sulfate was prescribed as an initial treatment and for patients with lower pain. Valproate sodium was used for patients whose pain was not improved by magnesium sulfate in previous sessions. If this was ineffective too, then DHE was considered. DHE was known to be more effective than magnesium sulfate and valproate sodium, and was prescribed for patients with higher pain. Based on self-reported pain scores, valproate sodium had a significant better pain improvement over magnesium sulfate (*P* = 0.03). However, self-reported pain score data do not show significant pain improvement between DHE and the other two drugs which could be the result of a small sample size.

### NIRS data

**Fig. 6:**
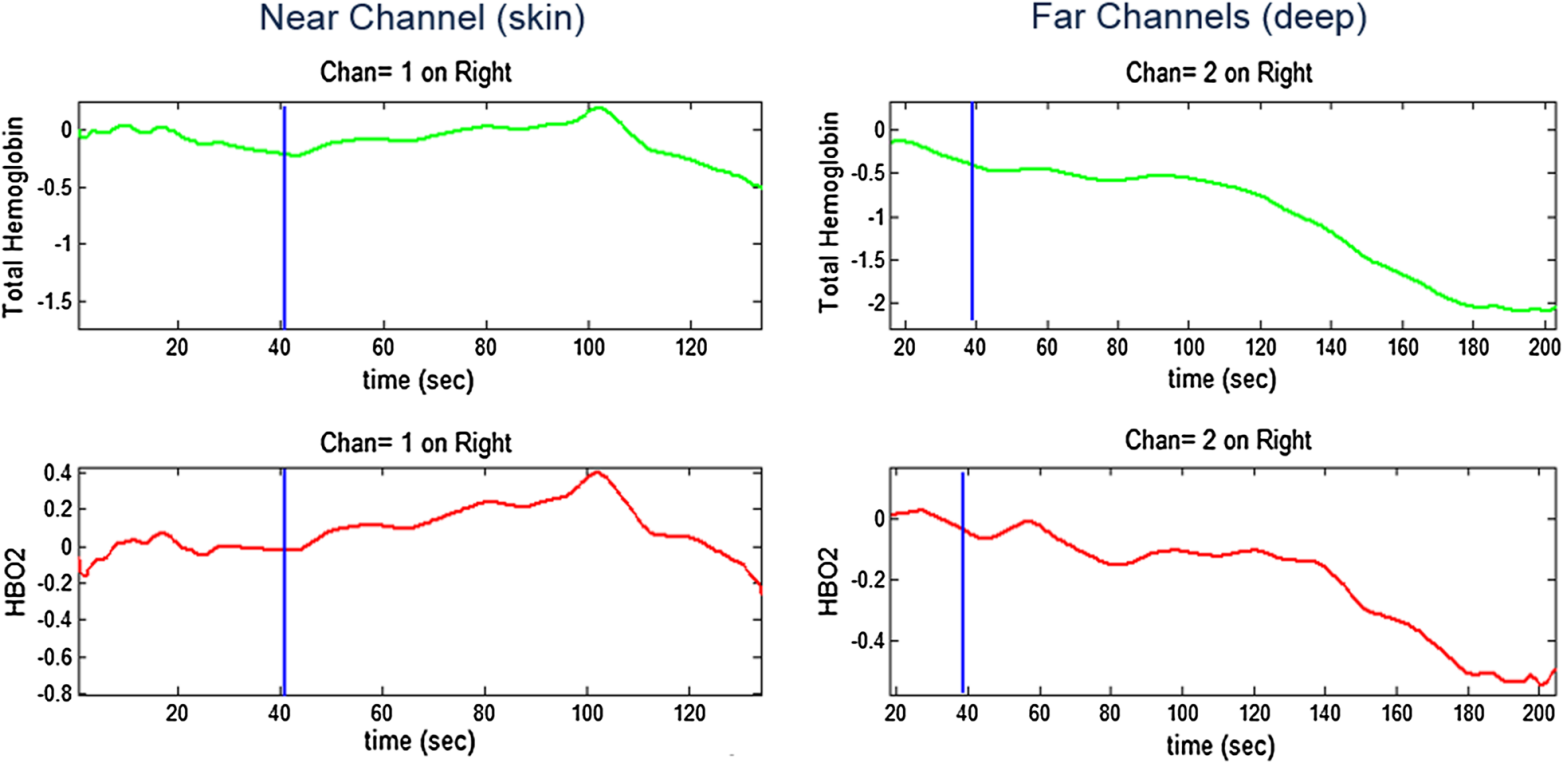
Sample of recorded NIRS data during medication infusion (DHE for this subject) on a migraine patient: near channel (on *left*) measures oxyhemoglobin (HbO_2_) and total hemoglobin (THb) changes during the infusion on the skin while far channels (on *right*) measure both skin and cortical activities. *Blue line* infusion time

Data were collected from six channels for each subject—one near channel measuring vascular response of the skin and two far channels measuring vascular response from cortex and skin together on each side. Changes in the detected light intensity were converted to concentration changes of oxyhemoglobin (HbO_2_) and deoxyhemoglobin (Hb) in the blood through Beer–Lambert law (Eqs. 1, 2). Figure 6 shows a sample of data reflecting changes in the total hemoglobin (THb) and oxyhemoglobin (HbO_2_) during the course of DHE infusion. Total hemoglobin is summation of oxyhemoglobin and deoxyhemoglobin and is a measure of total blood volume.

We examined the infusions’ effect on vascular reactivity. The extracted feature from the signal was slope of oxyhemoglobin (HbO_2_), deoxyhemoglobin (Hb) and total hemoglobin (THb) data before and after the infusion. After removing faulty signals, the remainder of the data included 18 magnesium sulfate, 10 valproate sodium and 6 DHE subjects. Due to small sample sizes and non-normality of the data, Mann–Whitney U test (Wilcox test) was used to check if the difference in slope change between medications were significant.

No significant differences were observed between different medications on near channels which measure skin vascularity responses. This suggests that alternations in skin blood flow and autonomic response induced by drug infusion were not different between drugs. However, the analyses on far left channels showed significant differences in both HBO_2_ and THB between valproate sodium and magnesium sulfate and between magnesium sulfate and DHE (*P* < 0.001) reflecting differences in cortical vascular reactivity among medications. In terms of slope changes in HbO_2_ and THb, we have the following order:

$${\text{Magnesium sulfate}} > 0 \,{ \sim }{\text{valproate sodium}} > {\text{DHE}}.$$

Positive slope change of THb for magnesium sulfate suggests that local blood volume has increased due to magnesium sulfate infusion and negative slope change for DHE shows decrease in blood volume by DHE infusion while for valproate sodium these changes are close to zero—no significant effect on blood volume.

Table 2 shows a summary of data and the changes in the HbO_2_, Hb and THb as measured by NIRS in an arbitrary unit for valproate sodium, magnesium sulfate and DHE as well as P values.

**Table 2:** Comparison of infusion vascular reactivity between medications on both near and far channels (mean and standard deviation values are only shown for far channels)

Far channel	HbO_2_		Hb		THb		HbO_2_		Hb		THb	
Drug	Valproate	Mg	Valproate	Mg	Valproate	Mg	DHE	Mg	DHE	Mg	DHE	Mg
Mean	−0.0003	0.004	0.003	0.01	−0.001	0.012	−0.001	0.004	0.013	0.02	−0.02	0.01
Standard deviation	0.001	0.005	0.009	0.027	0.002	0.012	0.001	0.005	0.083	0.027	0.03	0
P value (far)	0.008		0.22		0.0002		0.003		0.176		0.00001	
P value (near)	0.36		0.29		0.23		0.62		0.87		0.82	

### Cortical activity and skin response

In our experiments, near channels have penetration depth of 0.5 cm and measure alternations in skin vascular activity which are mostly caused by autonomic response. On the other hand, far channels capture up to 1.5 cm and measure cortical activities.

Our results on far channels showed that DHE decreased blood volume, magnesium sulfate increased blood volume and valproate sodium had no significant effect on the blood volume. The near channels, measuring skin vascularity, showed no significant differences between the medications indicating that the results are not due to alteration in skin vascular activity.

### Unilateral cortical response

Although data were collected from six channels on both sides of forehead, significant differences in cortical activities between medications were only observed on the left side. Different left and right side cerebral reactivity in migraineurs has been reported before in literature as well. For instance, [13] reports a smaller increase in right-sided total hemoglobin (THb) and significantly greater increase in left-sided total hemoglobin in migraineurs compared to volunteers. Moreover, some migraineurs have unilateral or side predominance pain which may also result in unilateral reactivity. A significantly greater increase in total hemoglobin (THb) and percent oxygen on the predominant migraine side compared with the non-predominant migraine side has been reported in [1].

However, the predominance side of pain (if existed at all) was not recorded in our data which makes it difficult to interpret.

### Correlation between cortical response and self-reported pain score

While these medications had significantly different effects on cortical vascular activities compared to each other, all three were effective in reducing the patients’ pain based on self-report. DHE, which is a well-known vasoconstriction, and MgSO4, which literature suggests causes cortical vasodilation, showed a decrease and an increase in blood volume, respectively; during our trial, yet they were both effective in improving pain and valproate sodium with no significant vascular effect had the best self-reported pain improvement among the medications.

No significant link was observed between changes in cortical vascular reactivity and improvement in subjects’ self-reported pain scores.

### Neuronal theory versus vascular theory

As explained briefly before there is no consensus about the underlying cause of migraine. It is possible that many factors may cause the class of headaches categorized as migraine. Neural and vascular theories are proposed as the main theories describing the pathophysiology of migraines [6]. Traditionally, it was thought that migraine resulted from vasoconstriction of cranial blood vessels leading to compensatory vasodilation. Although there is thought to be a decrease in cerebral blood flow in the acute phase that can cause the stereotypical aura, the pain in migraine is thought to be as a result of the increase in cerebral blood flow due to vasodilation of the middle meningeal artery. This increase in blood flow is dependent upon trigeminal and parasympathetic activation.

In several studies, magnetic resonance angiography (MRA) has been employed to verify this theory. The method has been used to measure arterial changes before and after infusion of different vasoactive drugs and also before and during migraine headache attacks. However, the results are contradictory. The most recent study found intracranial but not extracranial arterial dilatation on the headache side relative to the non-headache side [14] while another earlier study reported that the middle cerebral artery (intracranial) and middle meningeal artery (extracranial) were both dilated on the pain side versus the non-pain side [15], and another MRA study of drug-induced migraine attacks reported no side-to-side changes at all [16]. The difference can be due to different study designs and drug effects [17].

In another study, the effect of several vasodilators on meningeal arteries was investigated to find a connection between the effect of a substance on a meningeal vessel and its ability to artificially induce migraine. No clear correlation was found between the efficacy of a substance as a meningeal artery vasodilator in human and the ability to artificially induce migraine or the mechanism of action [18].

More recently, consensus has shifted toward a neural theory of migraine which considers the excessive neocortical cellular excitability as the main cause. According to this theory, neuronal hyperactivity will cause cortical spreading depression (CSD) and then CSD will trigger a migraine attack. CSD involves rapid depolarization of cortical neurons with cellular efflux of potassium, in turn triggering migraine [6].

## Conclusion

Based on our results, no significant correlation was found between cortical vascular changes and pain improvement. Both medications that have different vascular effect (Mg and DHE) have improved pain the same and valproate sodium which has no effect on vascularity had the best pain improvement. So our data suggest that vascular theory may not explain the pain improvement mechanism in migraine patients completely.

Our study had several limitations, which could be improved upon. We need a greater sample size of subjects (particularly DHE) to attain more statistically significant results. Subjects who had valproate sodium infusion can be interpreted as control subjects because the mechanism of the drug is such that it does not affect blood flow. However, it would be better to have control subjects with saline infusion only to control the effect of infusion itself on the data. Part of the significance of these results lies in the fact that the data were collected in a typical community pain clinic and not in a quiet research laboratory—speaking to the applicability of NIRS. Based on the work presented here, we believe that NIRS has the potential to be a useful tool in a clinical setting for assessing the vascular effects of various medications for headache and migraine. We believe that further technological improvement in NIRS hardware and signal analysis can make NIRS an even more useful tool for objective study and assessment of migraine. Furthermore, we hope that studies using NIRS can complement other measurements to facilitate the discussion about the underlying cause of the migraine headache.

## Abbreviations

DHE: dihydroergotamine
Hb: deoxygenated hemoglobin
HbO_2_: oxygenated hemoglobin
Mg: magnesium sulfate
NIRS: near-infrared spectroscopy
THB: total hemoglobin
